# Microbe Decoder uncovers functional traits of microbes in microbiome datasets

**DOI:** 10.1093/nar/gkag515

**Published:** 2026-05-21

**Authors:** Timothy J Hackmann, John P Parris, Rekha Seshadri, Christopher Lingga

**Affiliations:** Department of Animal Science, University of California, Davis, CA 95618, United States; Department of Animal Science, University of California, Davis, CA 95618, United States; DOE Joint Genome Institute, Lawrence Berkeley National Laboratory, Berkeley, CA 94720, United States; Department of Animal Science, University of California, Davis, CA 95618, United States

## Abstract

Microbe Decoder is a web server that predicts functional traits of microbes in microbiome sequencing datasets. Sequencing has revealed thousands of organisms in most ecosystems, but the functional traits of many organisms remain unclear. Existing tools can predict names of organisms or their genes, but they rarely predict concrete biological functions (e.g. fermentation or anaerobic growth). Microbe Decoder fills this gap using three complementary tools relying on taxonomy, metabolic networks, or machine learning. These tools accept either names or gene functions as inputs and are integrated into an easy-to-use web app. When tested against data for microbial isolates, Microbe Decoder showed good predictive performance (e.g. balanced accuracy of 0.85). When applied to datasets from the gut, sediment, and sea, it predicted shifts in functional traits over space and time. Microbe Decoder is designed for use with prokaryotes, with the goal of including eukaryotes in the future. By revealing functional traits of microbes in biological systems, Microbe Decoder will advance biology, medicine, and environmental science. Microbe Decoder is available at https://www.microbe-decoder.org/.

## Graphical Abstract

**Graphical Abstract:**
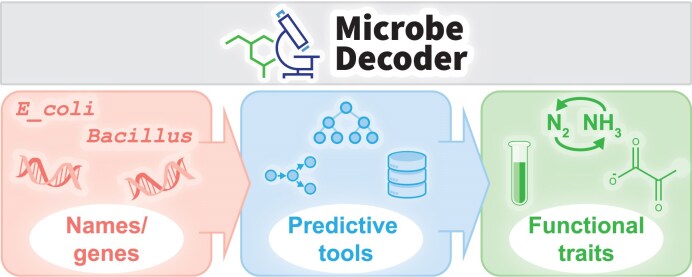

## Introduction

Microbiomes are part of every ecosystem on Earth, where they function in nutrient cycling [1, 2], energy capture [1, 2], and health of animal and plant hosts [3–5]. Despite their importance, the functions carried out by microbiomes usually cannot be attributed to specific organisms in the community. Most organisms in microbiomes have been detected with nucleic acid sequencing only [6–8], and they lack a close relative that can be studied in the lab and assigned functional traits. With sequencing of microbiomes growing exponentially, the problem is only growing more acute.

One solution is to use sequencing data to predict functional traits. It is now possible to use sequence data to predict presence of microbial taxa [9–11]. It is also possible to predict genes [12, 13] and annotate genes with functions [14–17]. Some tools go a step further and map genes to metabolic pathways [18–23]. While these predictions are useful, they are not concrete metabolic functions (e.g. fermentation) or other functional traits (e.g. anaerobic growth).

A new set of tools is now being developed to predict concrete functional traits. The tool FAPROTAX [24] predicts functional traits from names of taxa (taxonomy). This approach is straightforward, but it depends on query taxa having a close relative in an internal database. Other tools can predict functional traits from genes by using machine learning [25–27], an approach which does not depend directly on a database. Tools that map genes to metabolic pathways [18–23] can also predict functional traits if they depend on just one or a few enzymes (e.g. nitrogen fixation). More recently, we introduced a tool that makes predictions with two approaches (taxonomy and metabolic networks), but it focused on a single use case (fermentation) [28]. With further work, these tools could become a more routine part of microbiome analysis.

Here, we introduce Microbe Decoder, a web server that predicts microbial functional traits from a range of data types. It has three independent tools and puts them in an easy-to-use platform. We applied it to microbes from the gut, sediment, and sea. Its predictions closely agreed with available experimental data while also offering new insights. Microbe Decoder helps overcome a major bottleneck in microbiome analysis, advancing biology and fields beyond.

## Materials and methods

### Construction of database and tools

**Figure 1.**
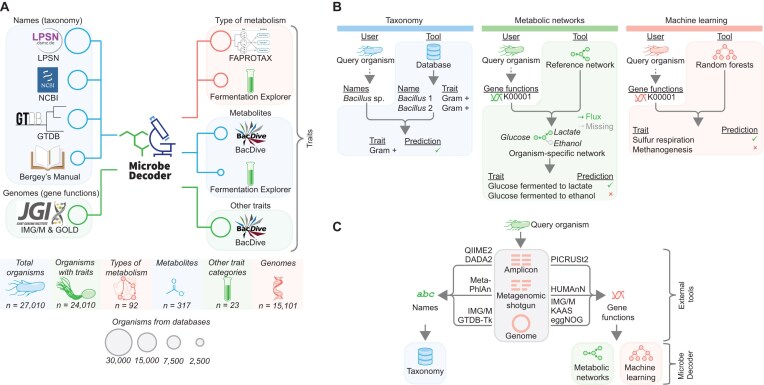
Microbe Decoder was constructed with an internal database and three predictive tools. (**A**) Construction of database. (**B**) Structure of tools. (**C**) Inputs into our tools, highlighting compatibility with external tools. Databases shown are LPSN [29], NCBI [30], GTDB [31], Bergey’s Manual [32], IMG/M [21], GOLD [33], FAPROTAX [24], Fermentation Explorer [28], and BacDive [35]. Tools shown are DADA2 [9], QIIME2 [36], MetaPhlAn [11], IMG/M [21], GTDB-Tk [37], PICRUSt2 [38], HUMAnN [19], KAAS [18], and eggNOG-mapper [15].

Microbe Decoder is built on a database of organism taxonomy, genomes, and functional traits from the literature (Fig. 1A). We constructed it by collecting names of validly published organisms from the LPSN [29]. This includes cultured bacteria and archaea from a large number of taxa (Supplementary Table S1). We then found matching records in other databases. For taxonomy, we used a total of four databases (LPSN [29], NCBI [30], GTDB [31], Bergey’s Manual [32]) in order to collect synonyms for these organisms. For genome information, we used the Genomes OnLine Database (GOLD) [33] to identify organisms with a sequenced genome, and the Integrated Microbial Genomes & Microbiomes (IMG/M) database [21] for gene functions (KEGG orthology IDs) [34]. For traits, we used three databases, including two developed by others (FAPROTAX [24] and BacDive [35]) and one we previously developed (Fermentation Explorer [28]). Some traits are reported by multiple databases, and all values are presented separately (with the database in parentheses). If one database reported multiple values for a single trait, all values were presented (in a list). Presenting information in this way allows users to identify areas of agreement and disagreement.

We constructed Microbe Decoder to include three predictive tools (Fig. 1B). The first relies on taxonomy, and the approach for prediction is from Fermentation Explorer [28] and inspired by FAPROTAX [24]. The input is organism names, which are matched to names of organisms in the database. The output is the probability of the trait, which is the fraction (0 to 1) of database organisms that are positive for it. The second tool is also from Fermentation Explorer [28] and relies on metabolic networks. The input is gene functions (KO IDs), which the tool maps to a reference network (all known reactions for a type of metabolism). It filters out any reactions that are not mapped, producing an organism-specific network (all reactions for a given organism). Using flux balance analysis, the tool determines if the organism-specific network has a path for metabolizing a substrate to an end product. The output is the metabolic flux, which is positive if a path is found. Reference networks have been updated from Fermentation Explorer [28] to increase the number of reactions by 3.1-fold. The third tool uses machine learning, and the approach for prediction is from MICROPHERRET [27]. The input is gene functions (KO IDs), which the tool uses as input into random forest classifier models. The models are trained using organisms in the database, with gene functions as predictors and trait presence as the response. The output is the probability of the trait, which is the fraction of trees (0–1) in the classifier giving a positive prediction. By default, tools consider a trait positive when probability > 0.5 or flux > 1. For reference, the flux of the substrate is set to 1000 (unitless).

We constructed our tools so the inputs can come from external tools popular in sequence analysis (Fig. 1C). Output from QIIME2 and four other external tools can be directly inputted into the taxonomy tool. Likewise, output from PICRUSt2 and four other external tools can be inputted into the metabolic networks and machine learning tools. These external tools, in turn, can handle amplicon, metagenomic shotgun, and whole-genome sequences. By accepting data from a range of external tools, our own tools can be used with a variety of samples and existing workflows.

### Evaluation of tools

We evaluated our tools with data for microbial isolates from the internal database or the literature. For the taxonomy tool, we randomly subsampled 30% of strains (organisms) from the internal database and used these as an evaluation dataset. We removed these strains from the database before running the tool, and the 70% of strains that remained were the training dataset. The training and evaluation datasets could share organisms from the same species, but not the same strain. We created additional datasets where the lowest taxonomic rank shared between training and evaluation data was genus, family, order, class, or phylum. To do this, we randomly selected 30% of the taxa for evaluation and ensured that the same groups did not appear in the training dataset. The metabolic networks tool was not trained using the database, and we evaluated performance with data for 100 model organisms from the literature (Supplementary Dataset S1). We identified these model organisms from MetaCyc [39], textbooks [40, 41], and the literature itself. For taxonomy and machine learning tools, we report the probability of predictions directly. For the metabolic networks tool, the probability was 1 if at least one product had flux > 1. We call this value the minimum flux and tested values below and above its default value. If the minimum flux was not met, the probability was 0.

We also evaluated our tools with data for microbial isolates characterized experimentally by our lab. These isolates are from the rumen, and we previously measured their formation of metabolites [28]. In this work, we tested them for anaerobic growth, aerobic growth, aerobic respiration, nitrate respiration, gram reaction, spore formation, and motility (Supplementary Fig. S1 and Supplementary Tables S2 and S3). We then predicted these traits with Microbe Decoder, FAPROTAX [24] (v. 1.2.12), DRAM (kb_DRAM v.0.1.2) [20], and BacDive-AI [25]. Input data (taxonomy, gene functions, or sequences) were downloaded using accession numbers in Supplementary Dataset S2. These data were processed and used to make predictions according to Supplementary Fig. S2. For Microbe Decoder, we removed all data for these isolates in the internal database before making predictions. All tools were run using default settings.

### Data for case studies

We demonstrated use of the web server using datasets for the gut of human infants [42], sediment in Winogradsky columns [43], and the water column of the Black Sea [44]. We downloaded sequence data using accession numbers in Supplementary Dataset S2. Data were processed externally with MetaPhlAn [11] (v. 4.1), DADA2 [9] (v. 1.34.0), PICRUSt2 [38] (v. 2.4.1), eggNOG-mapper [15] (v. 2.1.5), and GTDB-Tk [37] according to Supplementary Fig. S3. We used the output taxonomy or gene functions to make predictions with Microbe Decoder. Predictions were run using default settings, except for metagenome-assembled genomes (MAGs), where the “Enzymes must have all subunits” option was set to False. With this setting, the tool predicts an enzyme is present even if subunits are missing, which is common in MAGs.

We used output from Microbe Decoder and external tools to determine if functional traits differed over time and space. We calculated the abundance of each trait as the sum, across all taxa, of each taxon’s relative abundance multiplied by its probability of the trait. The relative abundance was from MetaPhlAn or DADA2 (Supplementary Fig. S3), and the probability was from Microbe Decoder. For metabolic networks, the probability was 1 if flux for at least one product was > 1 and 0 otherwise. We then tested if abundance differed over time or space using a nonparametric repeated measures analysis of variance. This was done using the ARTool [45] implementation in R (v. 0.11.2), art function, and model


\begin{eqnarray*}
A\sim f + (1|s),
\end{eqnarray*}

where *A* is the abundance of a trait, *f* is the factor (depth or age), and *s* is the experimental subject (infant or column). Means were separated using the emmeans package, emmeans function, and a Tukey’s test.

### Construction of the web server

Microbe Decoder was built as a web app using R. The Shiny package (v. 1.10.0) was used for basic functionality, the bslib package (v. 0.9.0) for the user interface, plumber package (v. 1.3.0) for the web application programming interface (API), and Plotly package (v. 4.10.4) for plots. For better network graphs, Plotly’s functions were extended to accept igraph [46] objects as inputs. For better phylogenetic trees, its functions were also extended to accept ggtree [47] objects. The networks tool uses the fbar package (v. 0.6.0) to solve network models with flux balance analysis. The machine learning tool uses the randomForest package (v. 4.7.1.1) to train and make predictions with random forest classifiers. The taxonomy tool was built using mostly base functions of R and the dplyr package (v. 1.1.4).

The app is available through a web server or for local download. The web server is run on Ubuntu Server (v. 24.04.3 LTS), which in turn runs the app inside Docker containers. Individual instances (containers) are launched and managed by a Node.js (v. 20.20.0) service, and nginx (v. 1.24.0) forwards web traffic to this service. Options for local download include an R Shiny app and Docker container image. The server is available at https://microbe-decoder.org/, the API at https://api.microbe-decoder.org/examples, and links for local download at https://github.com/thackmann/microbedecoder. This website is free and open to all users and there is no login requirement.

## Results

### Web server interface

**Figure 2.**
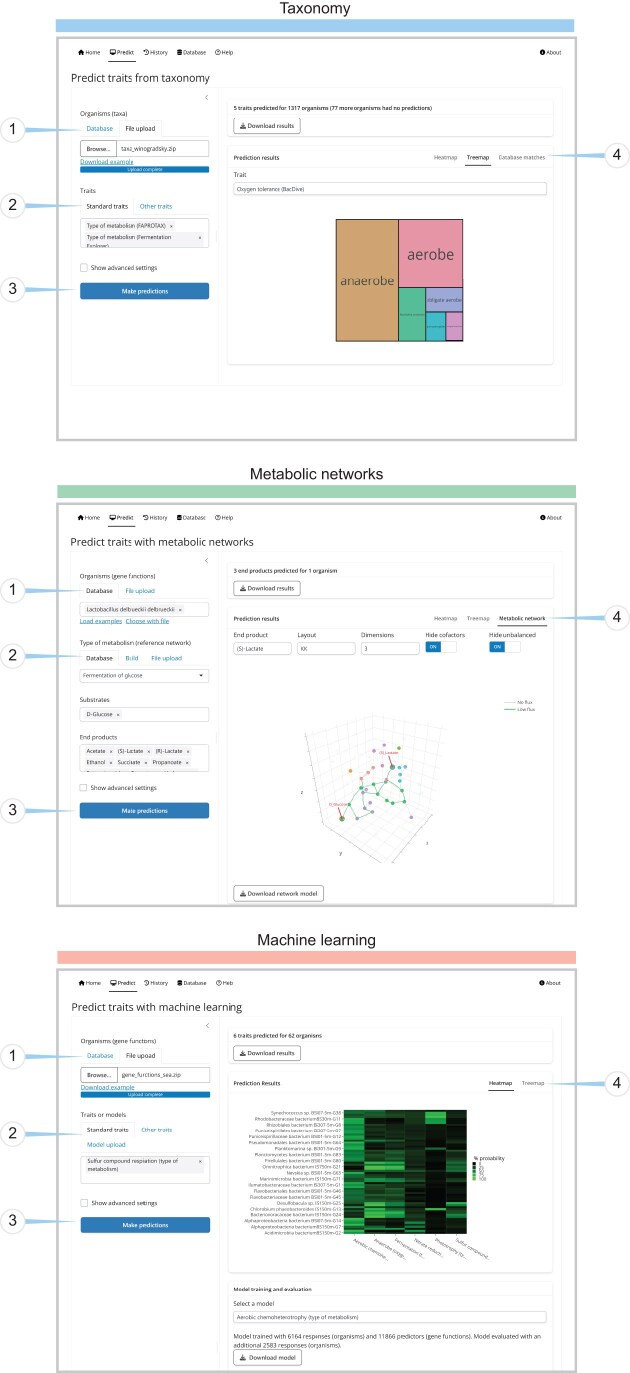
The web server of Microbe Decoder has multiple tools with an intuitive user interface. Highlighted are (i) options for data input, (ii) traits for prediction, (iii) button for running predictions, and (iv) options for interactive plots.

We designed Microbe Decoder with multiple tools for predicting microbial functional traits in an easy-to-use interface (Fig. 2). For each tool, the user selects input data by either (i) uploading a file or (ii) choosing organisms directly from the database. An uploaded file can come directly from popular tools for sequence analysis (see Fig. 1C), and example files can be downloaded. The user then selects functional traits to predict. After running predictions, the user can explore results in interactive plots, including heatmaps, treemaps, networks, and phylogenetic trees. Results are also available for download (as a .csv) and saved to the server for 30 days.

Many options are available for prediction. For the taxonomy tool, the user can select one of 9 traits. These traits vary little across strains within the same species (Supplementary Table S4). Custom traits can be defined with a query builder (Supplementary Fig. S4). Parameters for prediction, such as the system for taxonomy and strictness of matching, can also be specified. For the metabolic networks tool, the user can select the substrate, products, and reference network. The tool comes with 38 reference networks, and users can also build their own (Supplementary Fig. S4). For the machine learning tool, the user can select one of 30 pre-trained models. Users can also train their own machine learning model (Supplementary Fig. S4).

The server comes with other features. This includes utilities to search the database or download it, retrieve past jobs, and view video tutorials.

### Predictive performance

**Figure 3.**
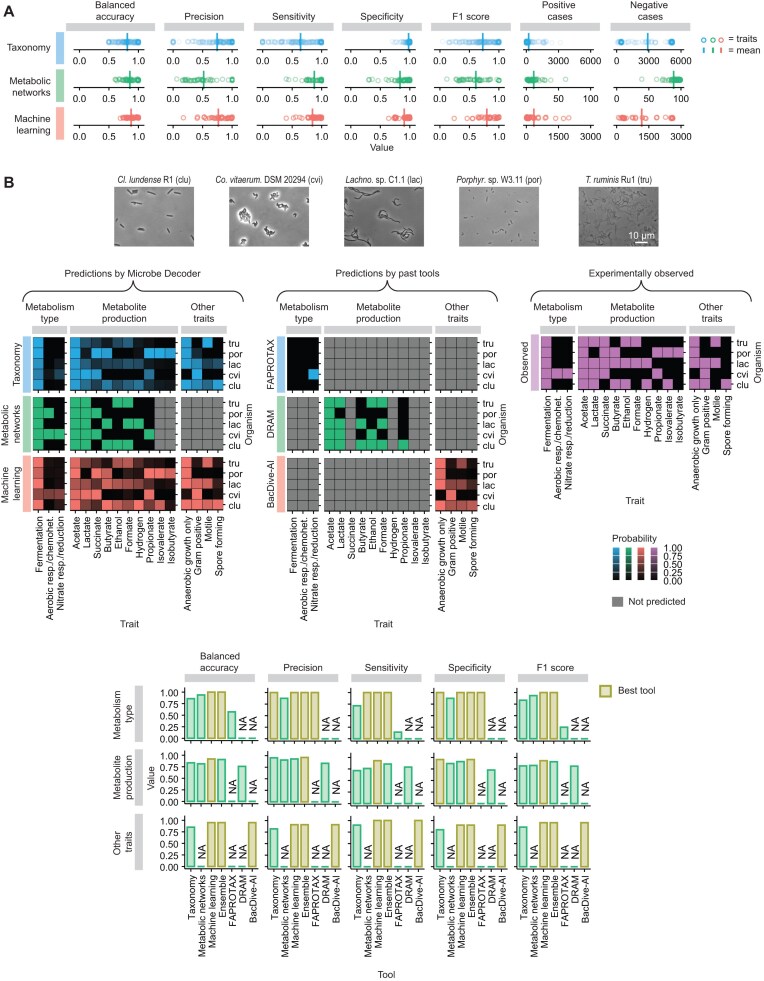
Microbe Decoder predicted functional traits of microbial isolates with high values of accuracy and other metrics. (**A**) Isolates from the internal database or literature. (**B**) Isolates from the rumen characterized by our lab. Included are predictions from FAPROTAX [24], DRAM [20], and BacDive-AI [25]. In panel (A), results for taxonomy and machine learning tools are for the rank of species, and results for the metabolic networks tool are for minimum flux of 1. Results for other taxonomic ranks are shown in Supplementary Fig. S5 and other minimum fluxes are in Supplementary Fig. S6. In panel (B), images of cells were taken using an Axio Scope.A1 microscope, 100× oil A-Plan objective with numerical aperture of 1.25, and Axiocam 305 mono camera (Zeiss, Munich, Germany). *Cl*. = *Clostridium*, chemohet. = chemoheterotrophy, *Co*. = *Corynebacterium, Lachno*. = *Lachnospiraceae, Porphyro*. = *Porphyromonadaceae*, resp. = respiration, and *vitaerum*. = *vitaeruminis*.

We evaluated Microbe Decoder’s predictive performance with microbial isolates with known functional traits. Overall, we found it had good predictive performance across tools and functional traits (Fig. 3A and Supplementary Dataset S3). Across traits, balanced accuracy averaged 0.81 for taxonomy, 0.86 for metabolic networks, and 0.88 for machine learning tools. Tools complemented each other; the machine learning tool, for example, had high precision and the taxonomy tool had high specificity. For taxonomy and machine learning tools, performance depended on the lowest taxonomic rank shared between training and evaluation datasets (Supplementary Fig. S5). Performance was highest when the rank was species or genus, moderate for family and order, and low for higher ranks. Performance of the metabolic networks tool was not sensitive to the minimum flux (default value of 1) (Supplementary Fig. S6). This evaluation was performed with isolates from the database or literature.

We also evaluated Microbe Decoder with isolates that were characterized by our lab, and predictive performance was again high (Fig. 3B and Supplementary Dataset S4). Further, it compared well with existing tools [20, 24, 25], having comparable or higher predictive performance while also covering more functional traits. The single tool that performed best was the machine learning tool of Microbe Decoder, and an ensemble prediction (an average of the taxonomy, metabolic networks, and machine learning tools) also performed well. Data for these isolates are available as example datasets on the server.

### Case studies

**Figure 4.**
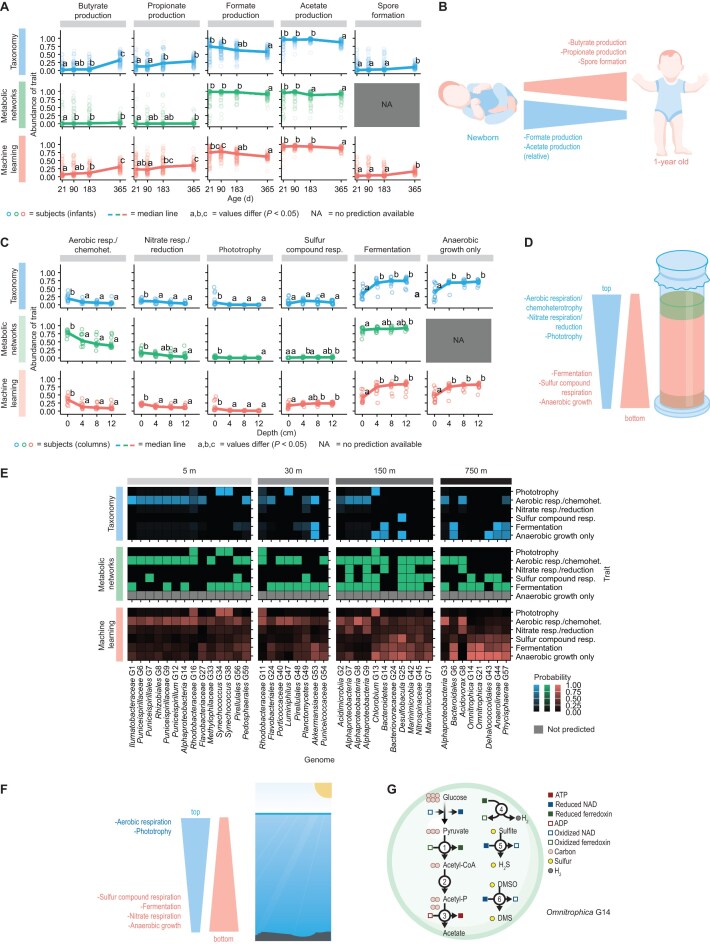
Microbe Decoder predicted functional traits of microbes in microbiomes from several environments. Predictions for microbes from infant gut (**A**) by age and (**B**) summarized graphically. Predictions for microbes from Winogradsky columns (**C**) by depth and (**D**) summarized graphically. Predictions for microbes from Black Sea (**E**) by depth, (**F**) summarized graphically, and (**G**) highlighting *Omnitrophica* G14, an organism from an uncultured phylum. Enzymes: 1, pyruvate-ferredoxin oxidoreductase (EC 1.2.7.1, 1.2.7.11); 2, phosphate acetyltransferase (EC 2.3.1.8); 3, acetate kinase (EC 2.7.2.1, 2.7.2.15); 4, membrane-bound hydrogenase (EC 1.12.7.2); 5, anaerobic sulfite reductase (EC 1.8.1.-); 6, anaerobic dimethyl sulfoxide reductase (EC 1.8.5.3) and Na^+^-transporting NADH:ubiquinone oxidoreductase (EC 7.2.1.1). Membrane-bound hydrogenase is shown in the cytoplasm but is actually located at the membrane. CoA = coenzyme A, chemohet. = chemoheterotrophy, DMS = dimethyl sulfide, DMSO = dimethyl sulfoxide, -P = phosphate, and resp. = respiration.

Microbe Decoder can make predictions for microbial isolates, but it is also designed to work with microbes in microbiome datasets. Toward this end, we illustrated its use with datasets for the infant gut [42], Winogradsky columns [43], and Black Sea [44] (Fig. 4). These datasets consist of organism names or gene functions from metagenomic shotgun sequences, amplicon sequences, and MAGs. All case studies are available as example datasets on the server.

When combined with abundance data from external tools, Microbe Decoder predicted clear shifts in functional traits over space and time. For the infant gut (Fig. 4A and B; Supplementary Fig. S7), it predicted an increase in butyrate-, propionate-, and spore-forming bacteria with age. It also predicted a decrease in formate- and acetate-producing bacteria. This is consistent with relative changes in these metabolites [48–51, 52] and spore-forming activity [51, 53], and it reflects how the gut matures with age. For Winogradsky columns (Fig. 4C and D), Microbe Decoder predicted a shift from aerobic respiration and nitrate respiration (at the top) to fermentation (at the bottom). Smaller shifts were also predicted for phototrophy and sulfur compound respiration. Aerobic respiration and nitrate respiration are energetically more favorable than other types of metabolism [54], and our predictions show these predominate at the top. For the Black Sea (Fig. 4E and F), it predicted a similar shift, but with sulfur compound respiration being particularly abundant at the bottom. This is consistent with data for the bottom being rich in reduced sulfur compounds [55]. Overall, there is good agreement between predictions and experimental observations or energetic theory.

Agreement among tools was generally high, but it depended on the dataset. It was highest for the infant gut, where organisms were closely related to those in the database (usually at the level of species) (Supplementary Fig. S8). Agreement was lowest for the Black Sea, where organisms were more distantly related (Supplementary Fig. S8). This pattern reflects that taxonomy and machine learning tools perform best with organisms related to those in the database.

Predictions do not just confirm known shifts, but they also reveal new insight into these systems. For the Black Sea, our results suggest that the organism *Omnitrophica* G14 can carry out sulfite and DMSO respiration (Fig. 4G). This organism belongs to a phylum with no cultured members, and DMSO respiration has not been reported for it [56], despite DMSO being found across aquatic systems [57].

Overall, Microbe Decoder predicted functional traits for a range of data types that were consistent with experimental observations while also extending knowledge. The predictions were accurate and easy to make, which provided more opportunity for discovery.

## Discussion

One of the grand challenges of microbiology is uncovering functions of microbes in the environment. Most microbes have not been cultured in the lab, and they are known from nucleic acid sequences alone [6–8]. Our goal with Microbe Decoder is to put prediction of functional traits within closer reach. It builds on previous tools [24–27], including our own [28], which rely on fewer approaches for prediction. Further, it is designed as an easy-to-use web server that complements current web servers in microbiology. One current server (BacDive [35]) used machine learning tools to predict traits of organisms in its database [35], and users can browse these pre-computed values. A second server (metaTraits [58]) allows users to browse pre-computed values and also submit genomes of single organisms for *de novo* prediction. Our server complements these resources by offering prediction approaches beyond machine learning, plus the ability to make de novo predictions for entire microbiome datasets.

There are several avenues for expanding Microbe Decoder. The current database includes cultured prokaryotes only, and future work should expand it to include both eukaryotes and uncultured prokaryotes. There is little functional information for the latter, but genomes are widely available [31]. Another direction is to predict interactions between organisms. At present, predictions are made for organisms in isolation, but organisms can interact in many ways, including exchanging metabolites [59]. A final direction is to provide better support for data for transcriptomes or proteomes. At present, predictions are based on all genes in the genome, but organisms do not express all genes.

As environmental microbiology has evolved, the bottlenecks for sequence analysis have shifted. With Microbe Decoder, our goal is to remove the current bottleneck of predicting functional traits of microbes. By understanding how microbes function, microbiologists can more fully focus on manipulating them and tackling the next grand challenge.

## Supplementary Material

gkag515_Supplemental_Files

## Data Availability

The Microbe Decoder web server is freely available at https://www.microbe-decoder.org/. All software discussed in this paper is open access and is freely available at https://github.com/thackmann/microbedecoder and https://doi.org/10.5281/zenodo.18651323. The data underlying this article are available in the article and in its online supplementary material.
