# Supplementary material for: Microbe Decoder uncovers functional traits of microbes in microbiome datasets
Source: Nucleic Acids Res. 2026 May 21;54(W1):W160–8. doi: 10.1093/nar/gkag515 (PMC13355083; doi:10.1093/nar/gkag515)
Supplement: gkag515_Supplemental_Files [file gkag515_supplemental_files.zip › Supplementary Data.pdf]

Supplementary Data for

Microbe Decoder uncovers functional traits of microbes in microbiome datasets

Timothy J. Hackmann[1]*, John P. Parris[1], Rekha Seshadri[2], and Christopher Lingga[1]

[1] Department of Animal Science, University of California, Davis, CA 95618 USA
[2] DOE Joint Genome Institute, Lawrence Berkeley National Laboratory, Berkeley, CA 94720 USA

* To whom correspondence should be addressed. Email: tjhackmann@ucdavis.edu

This PDF file includes:

Supplementary Fig. S1 to S8
Supplementary Tables S1 to S4
Supplementary References

Other supplementary material for this manuscript includes the following:

Supplementary Datasets S1 to S4

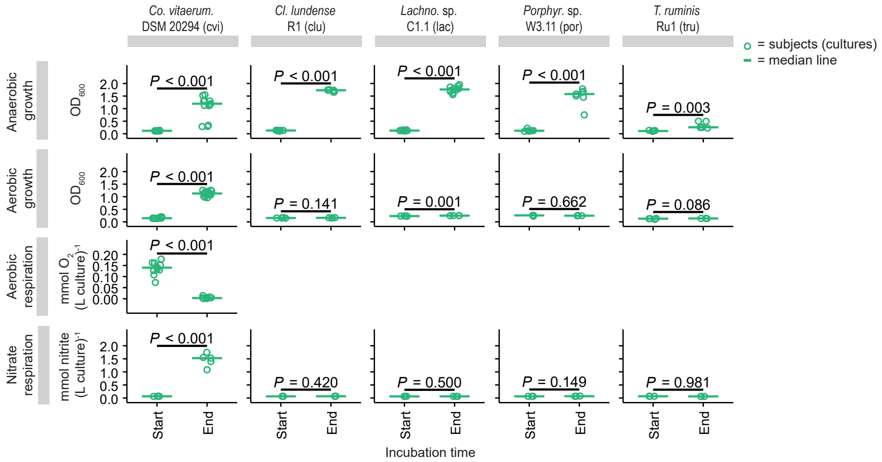

Supplementary Fig. S1. Characteristics of bacterial isolates from the rumen. Unless otherwise noted, tests were performed for cells grown anaerobically on PYG medium (Supplementary Table S3) according to ref. (1). To test for anaerobic and aerobic growth, cells were grown under normal conditions or under air. When grown under air, cysteine (a reducing agent) was withheld from media (Supplementary Table S3). For strains that grew under aerobic conditions, aerobic respiration was tested by measuring O_2 consumption with an O_2 electrode (Mettler Toledo 30266886). To test for nitrate respiration, cells were grown on nitrate media (Supplementary Table S3). At 0 and 6 days, 0.1 mL samples of culture were collected for measurement of nitrite. Samples were then combined with 2-mL test reagent (0.386 mM N-(1-naphthyl)ethylenediamine dihydrochloride, 28.9 mM sulfanilic acid, 1.5 M HCl) (3), incubated for 15 min, and color measured at 540 nm on a Molecular Devices M3 plate reader. Sodium nitrite was the standard. A total of $n = 3$ to 15 cultures were analyzed per organism and trait. Differences between start and end of incubation were determined using a paired, one-tailed *t*-test. At start of incubation, O_2 was normally measured under air. When measured under N_2 instead, the value was 0.087 (0.008 SEM) mmol O_2 L^{-1} culture ($n = 3$). At the end of the incubation, O_2 was normally measured under N_2. When measured under air instead, the value was 0.046 (0.001 SEM) mmol O_2 L^{-1} culture ($n = 2$). *Cl.* = *Clostridium*, *Co.* = *Corynebacterium*, *Lachno.* = *Lachnospiraceae*, *Porphyro.* = *Porphyromonadaceae*, and *vitaerum.* = *vitaeruminis*.

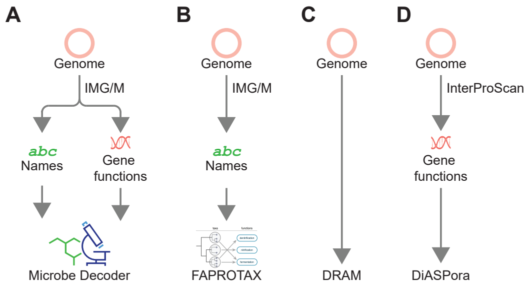

Supplementary Fig. S2. Prediction of functions for the rumen bacterial isolates dataset. Predictions were made with (A) Microbe Decoder, (B) FAPROTAX, (C) DRAM, and (D) DiASPora. Input data were downloaded from IMG/M (6) using accession numbers in Supplementary Dataset S2. For Microbe Decoder, the inputs were genome carts (containing taxonomy in NCBI format) and gene carts (containing gene functions). The output .csv files were used for further analysis. For FAPROTAX (3) (v. 1.2.12), the input was taxonomy put into FAPROTAX format. The output report.txt file was used for further analysis. The inputs for DRAM (4) were nucleotide .fasta files for the genomes. The output .html files were used for further analysis. The input for DiASPora (5) was a .tsv file containing Pfam IDs. This file was from InterProScan (7) (v. 5.65-97.0) and generated by running that tool on protein .fasta files for the genomes. The probabilities printed to the console were used for further analysis. All tools were run using default parameters, and DRAM was run on the KBase server (8) (as kb_DRAM v.0.1.2).

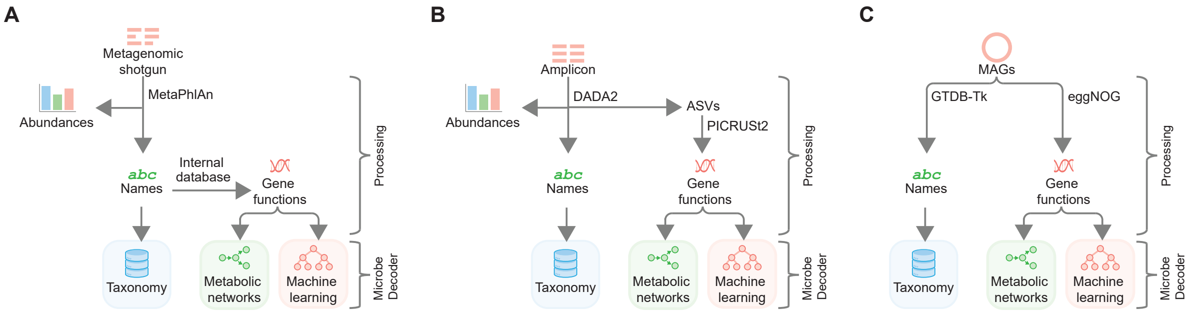

Supplementary Fig. S3. Processing of sequencing data for datasets for case studies. (A) Infant gut. (B) Winogradsky columns. (C) Black Sea. Sequences were downloaded from NCBI using accession numbers in Supplementary Dataset S2. For the infant gut dataset, data were processed using MetaPhlAn (9) (v. 4.1). The input was paired-end .fastq reads that were concatenated into a single file. Names (taxonomy) were in the output .tsv file. Gene functions were from the internal database of Microbe Decoder, and they were obtained by taking names from the .tsv file and selecting the corresponding organisms in our database. In an early analysis, we used gene functions from HUMAnN (10) (v. 4.0.0a1), but the number of predicted gene functions was low. Relative abundances of taxa were also in the .tsv file of MetaPhlAn, and they were used in statistical analysis. For the Winogradsky columns dataset, data were processed using DADA2 (11) (v. 1.34.0) and PICRUSt2 (12) (v. 2.4.1). The input into DADA2 were paired-end .fastq reads with primers removed. DADA2 was run with settings of the DADA2 pipeline tutorial (1.16) but with truncLen=c(175,150) and an updated version of the Silva reference database (13) (silva_nr99_v138.2_toGenus_trainset.fa.gz). Names (taxonomy) were from the seqtab.nochim object and saved to a .csv. The input into PICRUSt2 were amplicon sequence variants (ASVs) from DADA2. Gene functions were from the output KO_predicted.tsv. Relative abundances of taxa were also in the seqtab.nochim object of DADA2, and they were used in statistical analysis. For the Black Sea dataset, data were processed using eggNOG-mapper (14) (v. 2.1.5). The input was the protein .fasta files for genomes with >90% completeness and <5% contamination. Gene functions were from the output .tsv file (containing gene functions) was used for further analysis. Names (taxonomy) were from ref. (15), which previously analyzed genomes using GTDB-Tk (16). Unless otherwise noted, all tools were run using default settings.

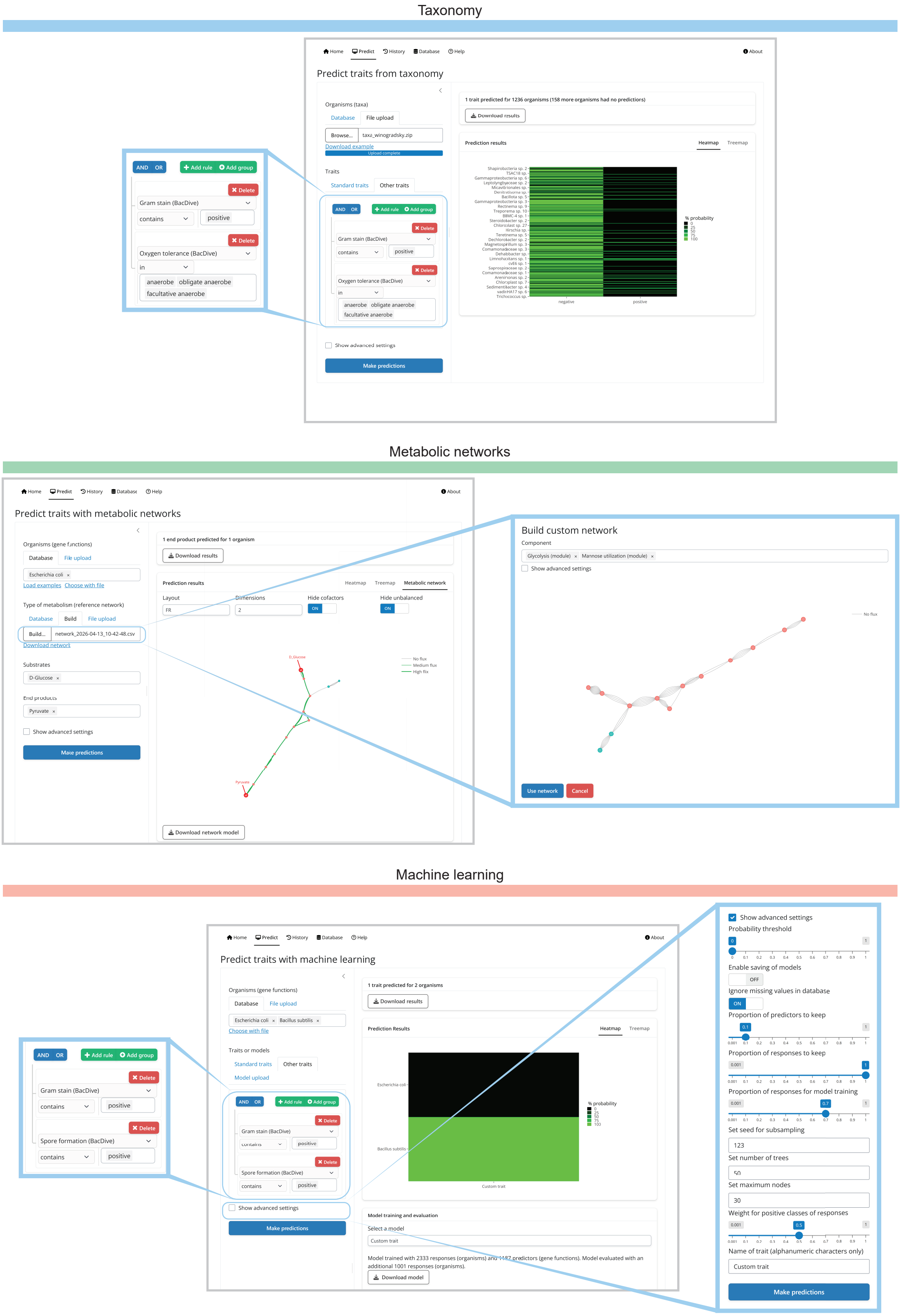

Supplementary Fig. S4. Microbe Decoder offers many options for prediction. This includes the ability to define custom traits (via Other traits), build custom metabolic networks (via Build), and train machine learning models (via Other traits).

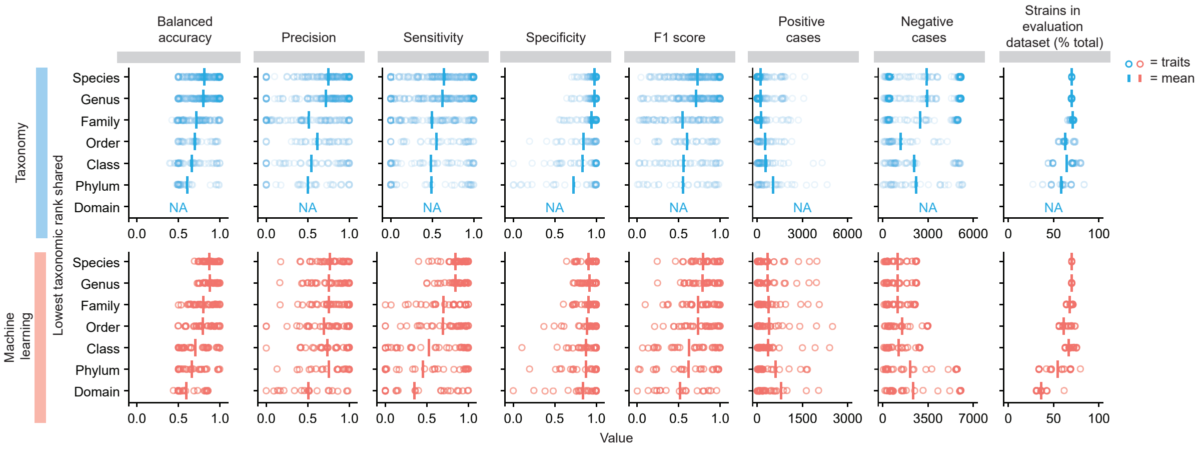

Supplementary Fig. S5. The performance of taxonomy and machine learning tools depends on lowest taxonomic rank shared between training and evaluation datasets. Strains in the evaluation dataset should be ~70%, but the value varied because taxa were sampled randomly and contained different numbers of strains. Results for the rank of species are also shown in Fig. 3.

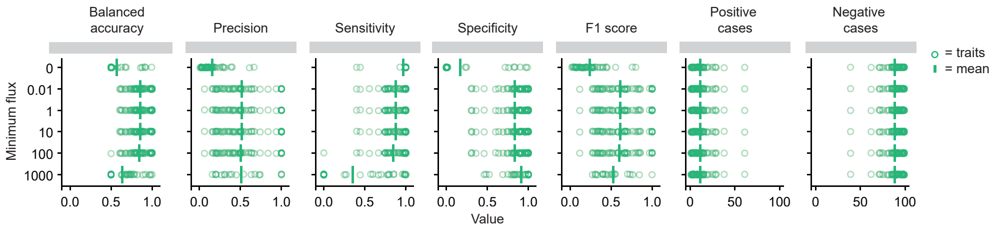

Supplementary Fig. S6. The performance of the metabolic networks tool is not sensitive to the value of minimum flux. Only low and high values affected performance. Results with minimum flux of 1 is also shown in Fig. 3.

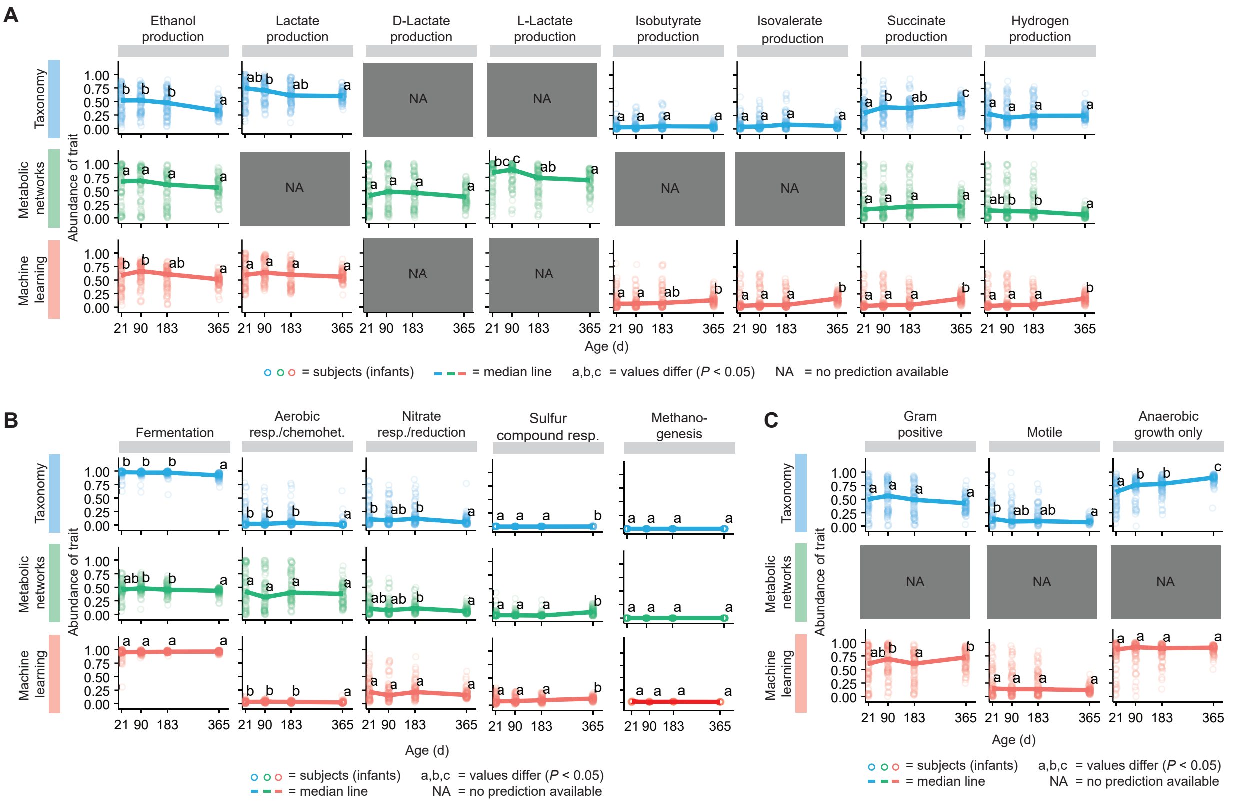

Supplementary Fig. S7. More functions predicted by Microbe Decoder for the infant gut. (**A**) Metabolite production. (**B**) Metabolism type. (**C**) Other traits. These are functions that changed little with age or inconsistently across tools. Chemohet. = chemoheterotrophy and resp. = respiration.

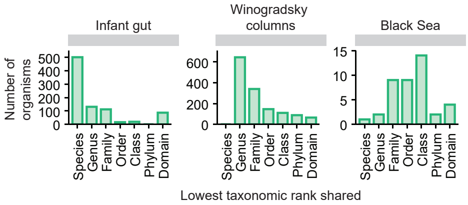

Supplementary Fig. S8. Organisms in sequencing datasets share taxonomic ranks with organisms in the internal database of Microbe Decoder. The degree of sharing was closest for the infant dataset and most distant for the Black Sea dataset.

Supplementary Table S1. The internal database of Microbe Decoder contains a large number of taxa.

Taxonomic rank	Number of taxa		
	Bacteria	Archaea	Total
Phylum	41	5	46
Class	108	15	123
Order	267	25	292
Family	723	46	769
Genus	4,339	183	4,522
Species	25,375	737	26,112
Strains	26,273	737	27,010

Supplementary Table S2. Other characteristics for bacterial isolates from the rumen.[1]

Organism[2]	Gram reaction		Spore formation[5]	Motility[5,6]
	Stain[3]	KOH test[4]		
Cl. lundense R1 (clu)	+ (violet)	+ (no thread)	+ (spherical, terminal)	-
Co. vitaerum. DSM 20294 (cvi)	+ (violet)	+ (no thread)	-	-
Lachno. sp. C1.1 (lac)	- (pink)	+ (no thread)	-	+ (swimming)
Porphyr. sp. W3.11 (por)	- (pink)	- (~3.5 cm thread)	-	-
T. ruminis Ru1 (tru)	- (pink)	- (~2.5 cm thread)	-	+ (swimming)

[1]Unless otherwise noted, tests were performed for cells grown anaerobically on PYG medium (Supplementary Table S3) according to ref. (1)

[2]*Cl. = Clostridium*, *Co. = Corynebacterium*, *Lachno. = Lachnospiraceae*, *Porphyro.= Porphyromonadaceae*, and *vitaerum. = vitaeruminis*.

[3]Tested following ref. (2)

[4]Tested following ref. (17). Values for this test supersede those for staining.

[5]Tested by observing cells with phase contrast microscopy (Axio Scope.A1 microscope, 100× oil A-Plan objective with numerical aperture of 1.25)

[6]*Lachnospiraceae* sp. C1.1 was not motile on PYG but was on Russell minimal media (Supplementary Table S3)

Supplementary Table S3. Media used in characterizing bacterial isolates from the rumen.

Component	Medium			
	PYG[1]	Aerobic PYG	Nitrate[2]	Russell minimal[3]
Glucose (mg/L)	5000	5000	1000	4000
K_2HPO_4 (mg/L)	2040	2040		292
KH_2PO_4 (mg/L)	40	40		292
Na_2HPO_4 (mg/L)			2000	
$(NH_4)_2SO_4$ (mg/L)				480
NaCl (mg/L)	80	80		480
$MgSO_4 \cdot 7H_2O$ (mg/L)	20	20		100
$CaCl_2 \cdot 2H_2O$ (mg/L)	10	10		64
Trypticase peptone (mg/L)[4]	5000	5000	20000	
Peptone (mg/L)[5]	5000	5000		
Yeast extract (mg/L)[6]	10000	10000		
Beef extract (mg/L)[7]	5000	5000		
$NaHCO_3$ (mg/L)	4000	4000		
Na_2CO_3 (mg/L)				4000
L-Cysteine $HCl \cdot H_2O$ (mg/L)	450			667
KNO_3 (mg/L)			1000	
Agar (mg/L)			1000	
Tween 80 (mL/L)	1	1		
Standard hemin solution (mL/L)[8]	10	10	10	
Russell hemin solution (mL/L)[9]				1
Vitamin K_1 solution (mL/L)[10]	0.2	0.2	0.2	
Pfennings heavy metal solution (mL/L)[11]				5
Russell vitamin solution (mL/L)[12]				10
VFA solution (mL/L)[13]				3.1
Headspace	CO_2	Air	N_2	CO_2

[1]Composition from ref. (18)
[2]Composition from ref. (19)
[3]Composition from ref. (20)
[4]Product 211921, BD
[5]Product 211677, BD
[6]Product 212750, BD
[7]Product LP0029, Oxoid
[8]0.5 g hemin and 0.4 g NaOH per liter
[9]0.1 g hemin, 0.28 g KOH, and 250 mL ethanol per liter
[10]5 mL vitamin K_1 and 950 mL ethanol per liter

[11]0.5 g EDTA disodium salt, 0.2 g $FeSO_4 \cdot 7H_2O$, 0.2 g $MnCl_2 \cdot 4H_2O$, 0.01 g $ZnSO_4 \cdot 7H_2O$, 0.03 g H_3BO_4, 0.02 g $CoCl_2 \cdot 6H_2O$, 0.001 g $CuCl_2 \cdot 2H_2O$, 0.002 g $NiCl_2 \cdot 6H_2O$, and 0.003 g $NaMoO_4 \cdot 2H_2O$ per liter

[12]0.1 g pyridoxamine dihydrochloride, 0.1 g pyridoxal hydrochloride, 0.1 g pyridoxine, 0.2 g riboflavin, 0.2 g thiamine HCl, 0.2 g nicotinamide, 0.2 g calcium pantothenate, 0.1 g lipoic acid, 0.01 g 4-aminobenzoic acid, 0.005 g folic acid, 0.005 g biotin, and 0.005 g coenzyme B_{12}, 1.1994 g K_2HPO_4, and 11.8123 g KH_2PO_4 per liter

[13]548 mL acetic acid, 194 mL propionic acid, 129 mL butyric acid, 32.3 mL valeric acid, 32.3 mL isovaleric acid, 32.3 mL isobutyric acid, and 32.3 mL 2-methylbutyric acid per liter

Supplementary Table S4. Most species with multiple strains have identical traits across those strains.

Trait	Cases with identical traits (A)[1]	Cases with non-identical traits (B)[1]	Total cases (A + B)[1]	Fraction identical [A/(A+B)]
Gram stain (BacDive)	461	0	461	1.00
Metabolites produced (Fermentation Explorer)	455	6	461	0.99
Metabolites utilized (Fermentation Explorer)	460	1	461	1.00
Motility (BacDive)	454	7	461	0.98
Oxygen tolerance (BacDive)	408	53	461	0.89
Pathogenicity (BacDive)	458	3	461	0.99
Spore formation (BacDive)	459	2	461	1.00
Type of metabolism (FAPROTAX)	461	0	461	1.00
Type of metabolism (Fermentation Explorer)	458	3	461	0.99

[1]Case refers to a species with multiple strains

Dataset S1 (separate file).
Model organisms used to evaluate the metabolic networks tool

Dataset S2 (separate file).
Accession numbers and metadata for sequence data analyzed in this study.

Dataset S3 (separate file).
Detailed statistics for evaluating Microbe Decoder with microbial isolates from the database or literature

Dataset S4 (separate file).
Detailed statistics for evaluating Microbe Decoder with microbial isolates characterized in our lab

Supplementary References

1. Hackmann,T.J. and Zhang,B. (2023) The phenotype and genotype of fermentative prokaryotes. *Sci Adv*, **9**, eadg8687.

2. Tindall,B.J., Sikorski,J., Smibert,R.A. and Krieg,N.R. (2007) Phenotypic characterization and the principles of comparative systematics. In Reddy,C.A., Beveridge,T.J., Breznak,J.A., Marzluf,G.A., Schmidt,T.M., Snyder,L.R. (eds), *Methods for General and Molecular Microbiology*. ASM Press, Washington, DC, USA, pp. 330–393.

3. Louca,S., Parfrey,L.W. and Doebeli,M. (2016) Decoupling function and taxonomy in the global ocean microbiome. *Science*, **353**, 1272–7.

4. Shaffer,M., Borton,M.A., McGivern,B.B., Zayed,A.A., La Rosa,S.L., Solden,L.M., Liu,P., Narrowe,A.B., Rodriguez-Ramos,J., Bolduc,B., *et al.* (2020) DRAM for distilling microbial metabolism to automate the curation of microbiome function. *Nucleic Acids Res*, **48**, 8883–8900.

5. Koblitz,J., Reimer,L.C., Pukall,R. and Overmann,J. (2025) Predicting bacterial phenotypic traits through improved machine learning using high-quality, curated datasets. *Commun Biol*, **8**, 897.

6. Chen,I.-M.A., Chu,K., Palaniappan,K., Ratner,A., Huang,J., Huntemann,M., Hajek,P., Ritter,S.J., Webb,C., Wu,D., *et al.* (2023) The IMG/M data management and analysis system v.7: content updates and new features. *Nucleic Acids Res*, **51**, D723–D732.

7. Jones,P., Binns,D., Chang,H.-Y., Fraser,M., Li,W., McAnulla,C., McWilliam,H., Maslen,J., Mitchell,A., Nuka,G., *et al.* (2014) InterProScan 5: genome-scale protein function classification. *Bioinformatics*, **30**, 1236–1240.

8. Arkin,A.P., Cottingham,R.W., Henry,C.S., Harris,N.L., Stevens,R.L., Maslov,S., Dehal,P., Ware,D., Perez,F., Canon,S., *et al.* (2018) KBase: The United States Department of Energy Systems Biology Knowledgebase. *Nat Biotechnol*, **36**, 566–569.

9. Blanco-Míguez,A., Beghini,F., Cumbo,F., McIver,L.J., Thompson,K.N., Zolfo,M., Manghi,P., Dubois,L., Huang,K.D., Thomas,A.M., *et al.* (2023) Extending and improving metagenomic taxonomic profiling with uncharacterized species using MetaPhlAn 4. *Nat Biotechnol*, **41**, 1633–1644.

10. Beghini,F., McIver,L.J., Blanco-Míguez,A., Dubois,L., Asnicar,F., Maharjan,S., Mailyan,A., Manghi,P., Scholz,M., Thomas,A.M., *et al.* (2021) Integrating taxonomic, functional, and strain-level profiling of diverse microbial communities with bioBakery 3. *Elife*, **10**, e65088.

11. Callahan,B.J., McMurdie,P.J., Rosen,M.J., Han,A.W., Johnson,A.J. and Holmes,S.P. (2016) DADA2: High-resolution sample inference from Illumina amplicon data. *Nat Methods*, **13**, 581–3.

12. Douglas,G.M., Maffei,V.J., Zaneveld,J.R., Yurgel,S.N., Brown,J.R., Taylor,C.M., Huttenhower,C. and Langille,M.G.I. (2020) PICRUSt2 for prediction of metagenome functions. *Nat Biotechnol*, **38**, 685–688.

13. Quast,C., Pruesse,E., Yilmaz,P., Gerken,J., Schweer,T., Yarza,P., Peplies,J. and Glöckner,F.O. (2013) The SILVA ribosomal RNA gene database project: improved data processing and web-based tools. *Nucleic Acids Res*, **41**, D590-6.

14. Cantalapiedra,C.P., Hernández-Plaza,A., Letunic,I., Bork,P. and Huerta-Cepas,J. (2021) eggNOG-mapper v2: functional annotation, orthology assignments, and domain prediction at the metagenomic scale. *Mol Biol Evol*, **38**, 5825–5829.

15. Cabello-Yeves,P.J., Callieri,C., Picazo,A., Mehrshad,M., Haro-Moreno,J.M., Roda-Garcia,J.J., Dzhembekova,N., Slabakova,V., Slabakova,N., Moncheva,S., *et al.* (2021) The microbiome of the Black Sea water column analyzed by shotgun and genome centric metagenomics. *Environ Microbiome*, **16**, 5.

16. Chaumeil,P.-A., Mussig,A.J., Hugenholtz,P. and Parks,D.H. (2019) GTDB-Tk: a toolkit to classify genomes with the Genome Taxonomy Database. *Bioinformatics*, **36**, 1925–1927.

17. Gregersen,T. (1978) Rapid method for distinction of gram-negative from gram-positive bacteria. *European J. Appl. Microbiol. Biotechnol.*, **5**, 123–127.

18. Zhang,B., Lingga,C., Bowman,C. and Hackmann,T.J. (2021) A new pathway for forming acetate and synthesizing ATP during fermentation in bacteria. *Appl Environ Microbiol*, **87**, e02959-20.

19. Holdeman,L.V., Cato,E.P. and Moore,W.E.C. (1977) Anaerobe laboratory manual 4th ed. Virginia Polytechnic Institute and State University, Blacksburg, VA.

20. Dai,X., Hackmann,T.J., Lobo,R.R. and Faciola,A.P. (2020) Lipopolysaccharide stimulates the growth of bacteria that contribute to ruminal acidosis. *Appl Environ Microbiol*, **86**.
